# Correlation Between Treatment‐Related Adverse Events and Efficacy of Camrelizumab in Combination With Apatinib in Patients With Unresectable Hepatocellular Carcinoma

**DOI:** 10.1002/cam4.70713

**Published:** 2025-03-23

**Authors:** Ting Zhang, Sicheng Du, Ying Zhang, Rongrui Liu, Juan Li, Chuanhua Zhao, Jianming Xu

**Affiliations:** ^1^ Chinese People's Liberation Army (PLA) Medical School Beijing China; ^2^ Department of Gastrointestinal Oncology, The First Medical Center Chinese PLA General Hospital Beijing China; ^3^ Department of Gastrointestinal Oncology, The Fifth Medical Center Chinese PLA General Hospital Beijing China

**Keywords:** angiogenesis inhibitor, hepatocellular carcinoma, immune checkpoint inhibitors, treatment‐related adverse events

## Abstract

**Background:**

The relationship between treatment‐related adverse events (TRAEs) and efficacy in patients receiving immune checkpoint inhibitors (ICIs) combined with anti‐angiogenic therapy remains unclear. This study aims to investigate the potential correlation between TRAEs and efficacy in patients with unresectable hepatocellular carcinoma (uHCC) treated with the combination of camrelizumab and apatinib.

**Methods:**

We conducted an analysis of efficacy and safety data obtained from 189 patients with uHCC enrolled in a phase II trial. All patients received intravenous camrelizumab 200 mg every 2 weeks and oral apatinib 250 mg once daily in 4‐week cycles. Efficacy was evaluated based on objective response rate (ORR), disease control rate (DCR), progression‐free survival (PFS), and overall survival (OS). We described the profiles of TRAEs and analyzed the correlation between TRAEs and treatment efficacy. To mitigate the impact of immortal time bias, landmark analysis and time‐dependent Cox regression analysis were employed to assess the correlation between immune‐related adverse events (irAEs) and survival outcomes.

**Results:**

As of March 10, 2021, irAEs of any grade were reported in 88 (46.6%) patients, with 17 (9.0%) patients experiencing grade 3–4 irAEs. The median onset time for any grade irAEs was 17.4 weeks. Apatinib‐related adverse events (AEs) of any grade were reported in 188 (99.5%) patients. Among them, 139 (73.5%) patients experienced any grade of apatinib‐related hypertension, while 65 (34.4%) patients had grade 3–4 hypertension. Patients who experienced irAEs exhibited significantly higher ORR and DCR, but the onset of irAEs occurred later than the time of PR or CR in 75.0% (30/40) of patients. Furthermore, in the landmark analysis and time‐dependent Cox regression analysis, no significant differences in survival outcomes were observed between patients with irAEs and those without. Notably, patients with apatinib‐related hypertension demonstrated better ORR (38.1% vs. 18.0%, *p* = 0.009) and DCR (84.2% vs. 60.0%, *p* < 0.001), as well as longer PFS (6.5 vs. 3.7 months, *p* = 0.001) and OS (23.0 vs. 15.1 months, *p* = 0.03).

**Conclusions:**

In this study, the occurrence of irAEs did not predict the efficacy of camrelizumab in combination with apatinib, likely due to the decreased incidence and delayed occurrence. On the other hand, apatinib‐related hypertension was associated with improved treatment efficacy.

**Abbreviations**
AE(s): adverse event(s)
AFP: A‐fetoprotein
BCLC: Barcelona clinic liver cancer
CR: complete response
CTCAE v4.03: Common Terminology Criteria for Adverse Events Version 4.03
DCR: disease control rate
DOR: duration of response
ECOG PS: Eastern Cooperative Oncology Group Performance Status
HBV: hepatitis B virus
HCV: hepatitis C virus
HFS: hand‐foot syndrome
HR: hazard ratio
HTN: hypertension
ICIs: immune checkpoint inhibitors
irAE(s): immune‐related adverse event(s)
IRC: independent review committee
LDH: lactate dehydrogenase
mRECIST: modified response evaluation criteria in solid tumors
NLR: neutrophil to lymphocyte ratio
NR: not reached
NSCLC: non‐small cell lung cancer
ORR: objective response rate
OS: overall survival
PD: progressive disease
PD‐1/PD‐L1: programmed death‐1/programmed death ligand‐1
PFS: progression‐free survival
PLA: People's Liberation Army
PR: partial response
RCCEP: reactive cutaneous capillary endothelial proliferation
RECIST v1.1: response evaluation criteria in solid tumors version 1.1
SD: stable disease
TACE: transarterial chemoembolization
TBIL: total bilirubin
TKIs: tyrosine kinase inhibitors
TRAEs: treatment‐related adverse events
TTR: time to response
(u)HCC: (unresectable) Hepatocellular carcinoma
ULN: upper limit of normal
VEGFR‐2: vascular endothelial growth factor receptor 2

## 1 Introduction

Primary liver cancer ranks as the sixth most commonly diagnosed cancer and the third leading cause of cancer‐related death worldwide according to the GLOBOCAN 2020 database [1]. Hepatocellular carcinoma (HCC) represents the predominant histological subtype, accounting for 75%–85% of cases [1]. A majority of patients with HCC are diagnosed at an advanced stage and require systemic therapy. Since 2017, immunotherapy has emerged as a major breakthrough following traditional sorafenib [2] targeted therapy for advanced HCC. CheckMate 040 [3] and KEYNOTE‐224 [4] have established the position of nivolumab and pembrolizumab as second‐line treatment options for advanced HCC, respectively. However, the subsequent phase III trial CheckMate 459 [5] and KEYNOTE‐240 [6] failed to demonstrate the clinical benefit of nivolumab as first‐line therapy compared to sorafenib and pembrolizumab as second‐line therapy compared to placebo.

Considering the limited efficacy of targeted agents and immune checkpoint inhibitors (ICIs) as monotherapy, in 2018, several phase I clinical trials exploring the combination of ICIs with anti‐angiogenic targeted therapy for advanced HCC exhibited promising results, significantly enhancing survival benefits for patients [7, 8, 9]. Subsequent multi‐center phase II–III randomized controlled studies involving programmed death‐1/programmed death ligand‐1 (PD‐1/PD‐L1) blockade combined with monoclonal antibody bevacizumab [10, 11] or small‐molecule tyrosine kinase inhibitors (TKIs) apatinib [12, 13] also demonstrated positive outcomes, thereby confirming the feasibility of combination therapy in clinical practice. Although the phase III study of pembrolizumab plus lenvatinib as first‐line therapy versus lenvatinib yielded negative results [14], causing considerable controversy worldwide, PD‐1/PD‐L1 blockade combined with bevacizumab or apatinib has now become the standard first‐line treatment for advanced HCC. Recently, transarterial chemoembolization (TACE), an important local treatment, combined with ICIs plus TKIs has been considered to be more effective than TACE monotherapy [15] and better than ICIs combined with TKIs [16].

Given the widespread implementation of PD‐1/PD‐L1 blockade combined with anti‐angiogenic targeted therapy in clinical practice, there is an urgent need to identify effective predictive biomarkers to facilitate the screening of potential beneficiaries. In certain solid tumors, such as non‐small cell lung cancer (NSCLC) and melanoma, the effectiveness of PD‐1/PD‐L1 blockade can be tentatively predicted by assessing PD‐L1 expression [17], microsatellite stability [18], and tumor mutational burden [19] in tumor tissues. However, a consensus regarding the predictive value of these biomarkers for HCC has not yet been reached [20]. Recent studies have suggested a correlation between the occurrence of immune‐related adverse events (irAEs) and improved efficacy in solid tumors, mainly in monotherapy [21, 22, 23]. Nevertheless, the data on this correlation are conflicting, as some studies demonstrate no such relationship [24, 25]. Presently, it remains uncertain whether this correlation persists when combined with anti‐angiogenesis targeted therapy, which poses a greater challenge to determine. Moreover, several studies have shown that patients experiencing anti‐angiogenesis‐related adverse reactions (AEs), such as hand‐foot syndrome (HFS), hypertension (HTN), and proteinuria, tend to have more favorable clinical outcomes [26, 27]. However, it remains unknown whether a similar phenomenon occurs when combined with ICIs.

Therefore, in this study, we present the profiles of irAEs and apatinib‐related specific AEs observed in patients receiving camrelizumab plus apatinib treatment within the RESCUE phase II trial. Additionally, we investigate the potential correlation between treatment‐related adverse events (TRAEs) and clinical outcomes.

## 2 Materials and Methods

### 2.1 Study Design and Patients

The data were collected from a non‐randomized, open‐label, phase II trial (RESCUE) conducted in 25 research centers in China. The study design and inclusion/exclusion criteria were reported in detail previously [12]. The primary inclusion criteria included patients with unresectable HCC (uHCC, unresectable or metastatic) confirmed by pathology, with at least one measurable lesion per RECIST v1.1, refractory to sorafenib or lenvatinib, or unable to afford sorafenib, a Child Pugh score of ≤ 6 (Child Pugh A), Barcelona Clinic Liver Cancer (BCLC) Stage B–C, and Eastern Cooperative Oncology Group performance status (ECOG PS) of 0 or 1. Patients with autoimmune diseases, those being treated with immunosuppressants or systemic steroid therapy, and those with brain metastasis were excluded. From March 13, 2018 to September 3, 2019, a total of 190 patients (70 first‐line patients and 120 second‐line patients) were enrolled in this trial. All patients received camrelizumab (Jiangsu Hengrui Pharmaceuticals Co. Ltd.) intravenously every 2 weeks at a dose of 200 mg (or 3 mg/kg for patients weighing less than 50 kg) and apatinib (Jiangsu Hengrui Pharmaceuticals Co. Ltd.) orally at a dose of 250 mg once daily in 4‐week cycles until disease progressed, unacceptable toxicity, or they reached the maximum 24 months of camrelizumab treatment. Patients who received at least one dose of camrelizumab plus apatinib were included in this analysis.

### 2.2 Assessment

Tumor assessment was conducted every 2 cycles (8 weeks) in the first 12 cycles and every 3 cycles thereafter. An independent review committee (IRC) performed the assessment according to the response evaluation criteria in solid tumors version 1.1 (RECIST v1.1) and modified response evaluation criteria in solid tumors (mRECIST) guidelines, which include complete response (CR), partial response (PR), stable disease (SD), and progressive disease (PD). This analysis primarily referred to the mRECIST guideline. Clinical outcomes evaluated included objective response rate (ORR, CR + PR), disease control rate (DCR, CR + PR + SD), progression‐free survival (PFS, the time from the first dose of study drugs to disease progression or death, whichever occurred first), and overall survival (OS, the time from the first dose of study drugs to all‐cause death). Additionally, the time to response (TTR, the time from the first dose of study drugs to CR or PR) and duration of response (DOR, the time from CR or PR to PD) were also used to assess the efficacy.

In this analysis, the TRAEs we analyzed were classified into two categories: (1) irAEs, which were associated with the administration of camrelizumab; (2) apatinib‐related specific AEs, including HTN, proteinuria, and HFS, which were related to the use of apatinib. AEs were collected until 30 days after the last administration, while serious adverse events and irAEs were collected until 90 days after the last administration or initiation of new anticancer treatment, whichever occurred first. AEs were graded according to the Common Terminology Criteria for Adverse Events Version 4.03 (CTCAE v4.03) of the National Cancer Institute.

### 2.3 Statistical Analysis

The categorical variables were described using numbers and percentages, and the difference between groups was compared using the chi‐square test or Fisher's exact test. The reverse Kaplan–Meier method was used to calculate the median follow‐up time. Survival curves were plotted using the Kaplan–Meier method, and the difference between the survival curves was evaluated using the log‐rank test. To mitigate time bias arising from the time dependence of irAEs, a landmark analysis was performed at 20 weeks (the median onset time of irAEs). Only patients with disease control or who were still alive at 20 weeks after the initiation of combination therapy were included for PFS or OS analysis, respectively. Additionally, a supplementary evaluation using a 6‐week landmark analysis was conducted. IrAEs occurring after the landmark date were disregarded. Furthermore, a time‐dependent Cox proportional regression model was employed to assess the prognostic value of irAE as a time‐varying variable on PFS and OS. The hazard ratio (HR) estimated through the time‐dependent Cox proportional regression model, known as adjusted HR, accounted for the immortal time bias. All statistical analyses were performed using SAS v9.4 and GraphPad Prism 9. A two‐sided *p* value < 0.05 was considered statistically significant.

## 3 Results

### 3.1 Patient Characteristics

**FIGURE 1:**
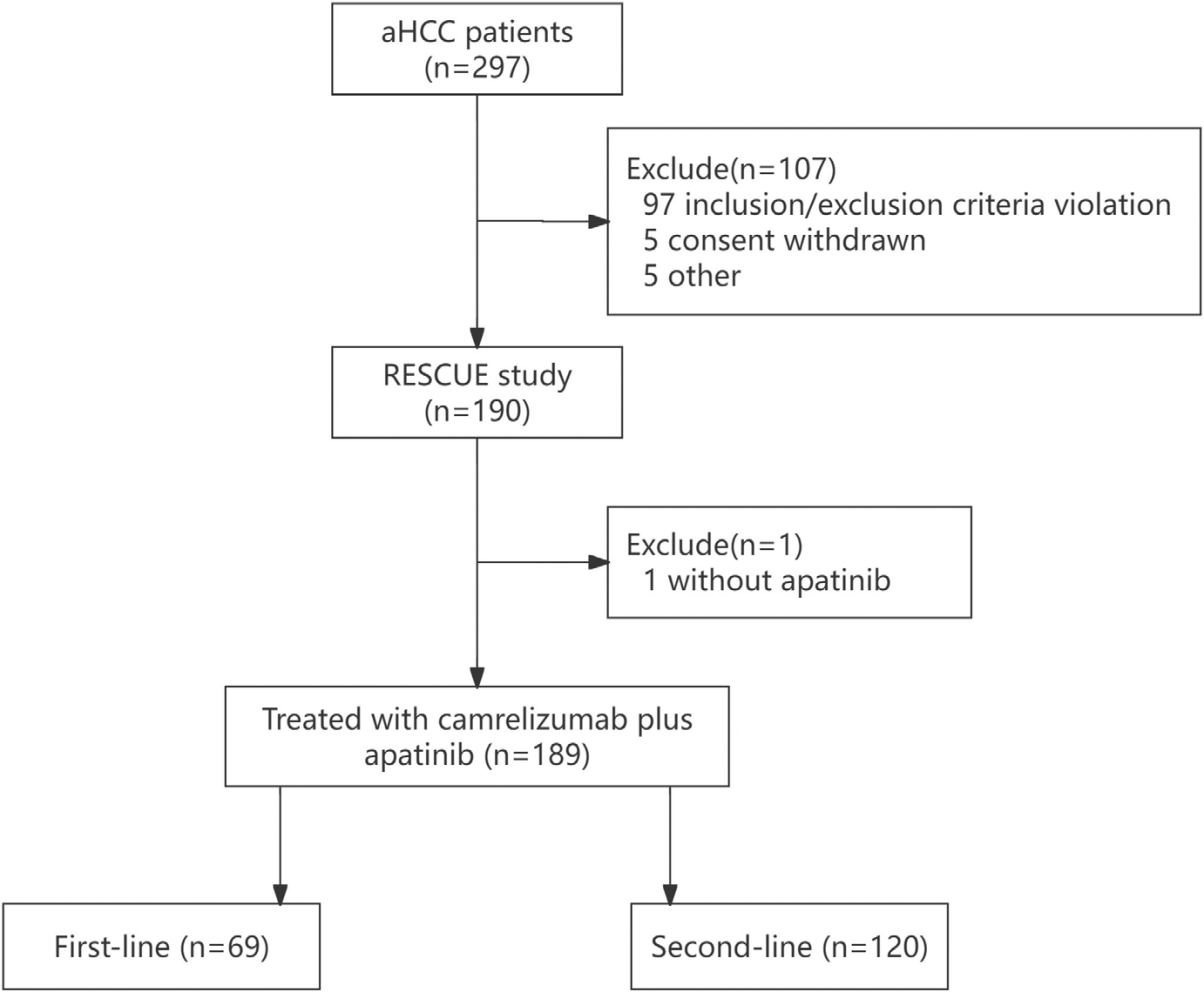
Screening chart of patients enrolled in this study.

A total of 189 patients were included in this analysis, including 69 patients in the first‐line treatment cohort and 120 in the second‐line treatment cohort (Figure 1). As of the data cutoff date, March 10, 2021, the median follow‐up time was 31.5 months (95% CI, 31.2–31.9). Among the 189 patients, the median age was 52 years. Of these, 114 (60.3%) had an ECOG score of 0, 161 (85.2%) had Child Pugh scores of 5, 34 (18.0%) had BCLC stage B, 149 (78.8%) had extrahepatic metastasis, 167 (88.4%) had hepatitis B virus (HBV) infection, 92 (48.7%) had A‐fetoprotein (AFP) ≥ 400 ng/mL, and 37 (19.6%) had lactate dehydrogenase (LDH) > 1 upper limit of normal (ULN). Baseline characteristics showed no statistically significant difference between the irAE group and the Non‐irAE group, except for the level of LDH. The proportion of patients with LDH > 1 ULN was higher in the Non‐irAE group (25.5% vs. 13.2%, *p* = 0.03). Details are shown in Table S1.

### 3.2 IrAEs Profile

Among the 189 patients receiving combination therapy, 122 irAEs occurred in 88 (46.6%) patients. Among the 88 patients with irAEs, 58 (65.9%) patients experienced 1 irAE, 27 (30.7%) patients experienced 2 irAEs, 2 (2.3%) patients experienced 3 irAEs, and 1 (1.1%) patient experienced 4 irAEs. The most frequently observed irAEs were reactive cutaneous capillary endothelial proliferation (RCCEP, *n* = 56, 29.6%), endocrine toxicity (*n* = 35, 18.5%), liver toxicity (*n* = 10, 5.3%), skin toxicity (*n* = 9, 4.8%), and pulmonary toxicity (*n* = 6, 3.2%). Nineteen grade 3–4 irAEs were observed in 17 (9.0%) patients, with hepatotoxicity (*n* = 9, 4.8%), endocrine toxicity (*n* = 3, 1.6%), pulmonary toxicity (*n* = 2, 1.1%), and RCCEP (*n* = 2, 1.1%) being the most common. One patient died from pulmonary toxicity (pneumonitis). Twenty‐three (12.2%) patients discontinued the combination therapy of camrelizumab and apatinib due to TRAEs, and 137 (72.5%) patients encountered treatment interruption or modification because of TRAEs. Twenty patients received systemic steroid therapy due to irAEs, and 16 of them used steroids for more than 30 days. The median onset time of any grade irAEs was 17.4 (range, 1.0–108.0) weeks, with skin irAEs having the shortest median onset time (15.4 weeks), while bladder irAEs had the longest median onset time (59.1 weeks). Additionally, the median onset time for RCCEP was 22.9 (range, 1.0–75.3) weeks, and for irAEs other than RCCEP, it was 18.6 (range, 1.7–108.0) weeks. Further details were provided in Table 1.

**TABLE 1:** Immune‐related adverse events in 189 patients treated with camrelizumab and apatinib.

irAEs	Total, *n* (%)	Grade 1–2, *n* (%)	Grade 3–5, *n* (%)	Systemic steroid therapy, *n*	Weeks to onset, median (range)
Any	88 (46.6)	70 (37.0)	18 (9.5)	20	17.4 (1.0–108.0)
RCCEP	56 (29.6)	54 (28.6)	2 (1.1)	0	22.9 (1.0–75.3)
Endocrine	35 (18.5)	32 (16.9)	3 (1.6)	1	16.1 (4.0–108.0)
Hypothyroidism	18 (9.5)	18 (9.5)	0	0	
Hyperthyroidism	3 (1.6)	3 (1.6)	0	0	
Thyroiditis	1 (0.5)	1 (0.5)	0	0	
Hyperglycemia	7 (3.7)	6 (3.2)	1 (0.5)	0	
Diabetes mellitus	4 (2.1)	3 (1.6)	1 (0.5)	0	
Ketoacidosis	1 (0.5)	0	1 (0.5)	0	
Lymphocytic hypophysitis	1 (0.5)	1 (0.5)	0	1	
Hepatobiliary	10 (5.3)	1 (0.5)	9 (4.8)	9	20.2 (2.1–95.7)
Immune‐mediated hepatitis	4 (2.1)	0	4 (2.1)	4	
Drug‐induced liver injury	1 (0.5)	0	1 (0.5)	1	
Hepatic function abnormal	5 (2.6)	1 (0.5)	4 (2.1)	4	
Skin	9 (4.8)	8 (4.2)	1 (0.5)	1	15.4 (1.7–61.7)
Rash	7 (3.7)	7 (3.7)	0	0	
Eczema	1 (0.5)	1 (0.5)	0	0	
Drug eruption	1 (0.5)	0	1 (0.5)	1	
Pulmonary	6 (3.2)	3 (1.6)	3 (1.6)	6	26.0 (8.0–70.1)
Pneumonitis	4 (2.1)	1 (0.5)	3 (1.6)	4	
Interstitial lung disease	1 (0.5)	1 (0.5)	0	1	
Immune‐mediated pneumonia	1 (0.5)	1 (0.5)	0	1	
Bladder	4 (2.1)	3 (1.6)	1 (0.5)	4	55.6 (22.4–68.9)
Cystitis hemorrhagic	2 (1.1)	2 (1.1)	0	2	
Immune‐mediated cystitis	2 (1.1)	1 (0.5)	1 (0.5)	2	
Gastrointestinal	1 (0.5)	1 (0.5)	0	1	33.4 (33.4–33.4)
Immune‐mediated enterocolitis	1 (0.5)	1 (0.5)	0	1	
Others	2 (1.1)	1 (0.5)	1 (0.5)	1	31.4 (17.7–45.1)
Fatigue	1 (0.5)	1 (0.5)	0	0	
Platelet count decreased	1 (0.5)	0	1 (0.5)	1	

### 3.3 Correlation Between irAEs and Efficacy

**FIGURE 2:**
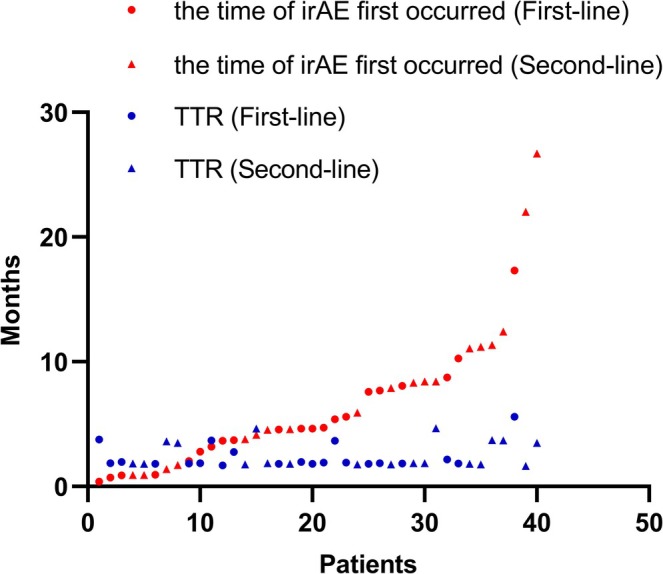
Scatter plot depicting the time of irAE first occurrence and the time to response (TTR) in the 40 patients with responses in the irAE group.

At the time of analysis, the ORR and DCR in the overall population were 32.8% (62/189) and 77.8% (147/189), respectively. Among the 88 patients with irAEs, 5 (5.7%) achieved CR, 35 (39.8%) experienced PR, and 37 (42.0%) had SD. In contrast, among the 101 patients without irAEs, CR, PR, and SD were observed in 3 (3.0%), 19 (18.8%), and 48 (47.5%) patients, respectively. Patients with irAEs demonstrated significantly higher ORR (45.5% vs. 21.8%, *p* = 0.001) and DCR (87.5% vs. 69.3%, *p* = 0.003) compared to those without irAEs. Consistent results were observed in both the first‐line and second‐line patients, regardless of whether the tumor response was evaluated according to the RECIST v1.1 or mRECIST (Table 2). Among patients with tumor responses, the median TTR was 1.9 (range, 1.4–11.1) months, and the median DOR was 9.3 (range, 1.7–17.6) months. There was no statistically significant difference in TTR between the irAE group and Non‐irAE group (1.9 vs. 1.8 months, *p* = 0.34), while the DOR was higher in the irAE group (11.9 vs. 7.7 months, *p* = 0.01). However, for patients with tumor response in the irAE group, the median onset time of irAE and median TTR were 4.7 (range, 0.4–26.7) months and 1.9 (range, 1.6–5.6) months, respectively, with the onset of irAEs occurring later than the time of PR or CR in 75.0% (30/40) of patients (Figure 2).

**TABLE 2:** Correlation between tumor response and irAEs in 189 patients treated with camrelizumab and apatinib per mRECIST and RECIST v1.1.

	IRC per mRECIST				IRC per RECIST v1.1			
	Total, *n* (%)	irAE, *n* (%)	Non‐irAE, *n* (%)	*p*	Total, *n* (%)	irAE, *n* (%)	Non‐irAE, *n* (%)	*p*
Best overall response				0.001				< 0.001
CR	8 (4.2)	5 (5.7)	3 (3.0)		3 (1.6)	3 (3.4)	0	
PR	54 (28.6)	35 (39.8)	19 (18.8)		48 (25.4)	34 (38.6)	14 (13.9)	
SD	85 (45.0)	37 (42.0)	48 (47.5)		95 (50.3)	40 (45.5)	55 (54.5)	
PD	37 (19.6)	11 (12.5)	26 (25.7)		38 (20.1)	11 (12.5)	27 (26.7)	
NE	5 (2.6)	0	5 (5.0)		5 (2.6)	0	5 (5.0)	
ORR	62 (32.8)	40 (45.5)	22 (21.8)	0.001	51 (26.9)	37 (42.0)	14 (13.9)	< 0.001
First‐line	32 (46.4)	21 (61.8)	11 (31.4)	0.01	24 (34.8)	17 (50.0)	7 (20.0)	0.009
Second‐line	30 (25.0)	19 (35.2)	11 (16.7)	0.02	27 (22.5)	20 (37.0)	7 (10.6)	0.001
DCR	147 (77.8)	77 (87.5)	70 (69.3)	0.003	146 (77.2)	77 (87.5)	69 (68.3)	0.002
First‐line	56 (81.2)	32 (94.1)	24 (68.6)	0.02	55 (79.7)	32 (94.1)	23 (65.7)	0.003
Second‐line	91 (75.8)	45 (83.3)	46 (69.7)	0.08	91 (75.8)	45 (83.3)	46 (69.7)	0.08

**FIGURE 3:**
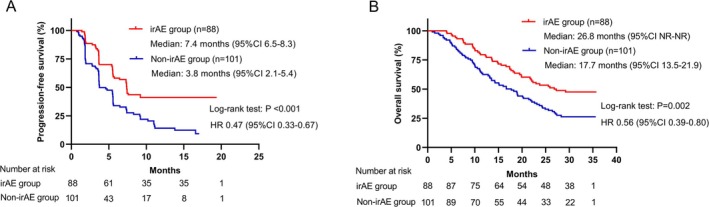
Comparisons in survival between the irAE group and Non‐irAE group. Kaplan–Meier curves of (A) progression‐free survival and (B) overall survival.

**FIGURE 4:**
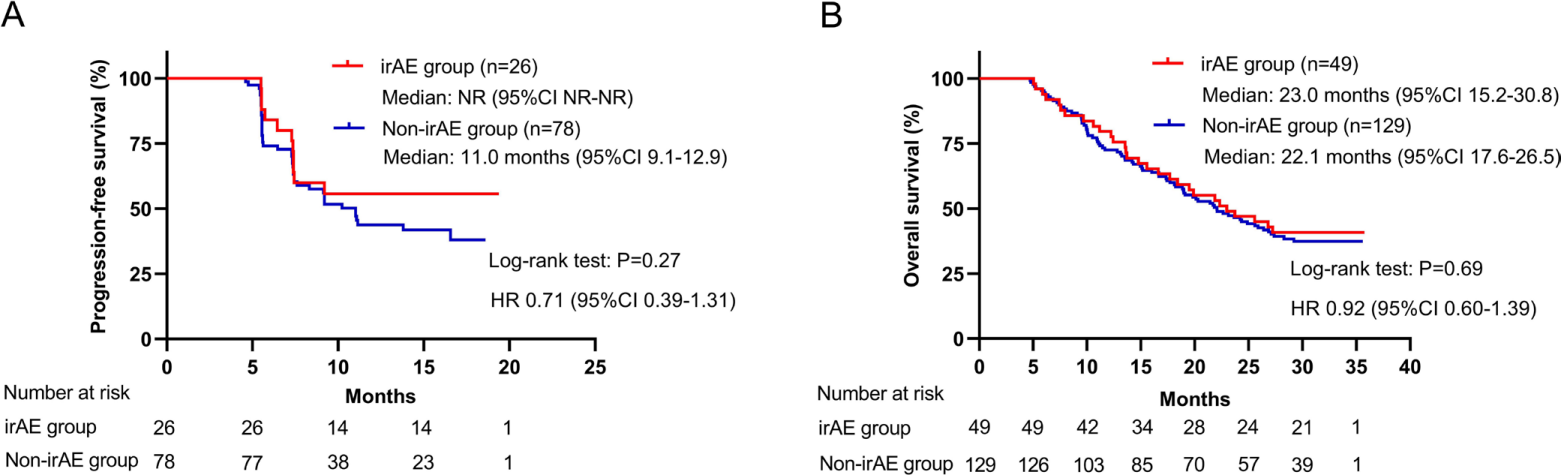
Kaplan–Meier curves with 20‐week landmark analysis for (A) progression‐free survival and (B) overall survival in patients with or without irAE.

The median PFS was 5.6 months (95% CI, 4.9–6.2) and the median OS was 21.7 months (95% CI, 18.1–25.2) in the overall population. Patients with irAEs had significantly prolonged median PFS (7.4 vs. 3.8 months, *p* < 0.001) and median OS (26.8 vs. 17.7 months, *p* = 0.002) compared to those without irAEs (Figure 3). Consistent results were observed in both first‐line and second‐line patients (Figure S1). However, it should be noted that patients with longer treatment durations were more likely to experience irAEs. To mitigate the time bias resulting from different treatment durations, we performed a landmark analysis and time‐dependent Cox regression analysis. In the 20‐week landmark analysis, there was no significant difference in PFS (not reached [NR] vs. 11.0 months, HR 0.71, *p* = 0.27) and OS (23.0 vs. 22.1 months, HR 0.92, *p* = 0.69) between the irAE group and Non‐irAE group (Figure 4). Similar findings were observed in the 6‐week landmark analysis (Figure S2). When irAE was included in the time‐dependent Cox regression analysis as a time‐varying covariate, no statistically significant positive effect of irAEs on PFS (adjusted HR 0.96, *p* = 0.85) and OS (adjusted HR 0.74, *p* = 0.12) was observed. These results (PFS: adjusted HR 1.08, *p* = 0.74; OS: adjusted HR 0.73, *p* = 0.12) remained consistent after adjusting for potential confounding factors (ECOG PS, age, gender, number of treatment lines, NLR, AFP, and LDH).

Given that RCCEP was a common and unique irAE associated with camrelizumab treatment, we conducted an analysis excluding RCCEP to investigate the correlation between irAEs and survival. Baseline characteristics showed no statistically significant difference between the irAE (excluding RCCEP) group and the Non‐irAE (excluding RCCEP) group, except for the level of LDH (Table S2). Patients with irAEs (excluding RCCEP) demonstrated longer PFS (7.4 vs. 5.5 months, *p* = 0.01) and OS (26.8 vs. 19.0 months, *p* = 0.01) than those without irAEs (excluding RCCEP) (Figure S3A,B). However, in the 20‐week landmark analysis, there were no significant differences in PFS (NR vs. 11.0 months, *p* = 0.54) and OS (21.9 vs. 22.6 months, *p* = 0.92) between the irAE (excluding RCCEP) group and Non‐irAE group (excluding RCCEP) (Figure S3C,D). Similarly, there was no difference in the univariate (PFS: adjusted HR 1.06, *p* = 0.82; OS: adjusted HR 0.78, *p* = 0.26) and multivariate (PFS: adjusted HR 1.16, *p* = 0.57; OS: adjusted HR 0.80, *p* = 0.32) time‐dependent Cox regression analysis, which was consistent with the results observed for all irAEs.

We further analyzed the correlation between the types of irAEs and efficacy. There is no difference between the RCCEP group and Non‐RCCEP group in baseline characteristics (Table S2). Patients with RCCEP had significantly higher ORR (48.2% vs. 26.3%, *p* = 0.003) and DCR (87.5% vs. 73.7%, *p* = 0.04), as well as longer PFS (7.4 vs. 5.5 months, *p* < 0.001) and OS (NR vs. 19.0 months, *p* = 0.003), compared to those without RCCEP (Figure S4A,B). In the 20‐week landmark analysis, there was also no significant difference in PFS (NR vs. 11.0 months, *p* = 0.19) and OS (26.8 vs. 22.1 months, *p* = 0.62) between the RCCEP group and Non‐RCCEP group (Figure S4C,D). In the time‐dependent Cox regression analysis, no positive prognostic effect of RCCEP on PFS (adjusted HR 0.68, *p* = 0.22) and OS (adjusted HR 0.64, *p* = 0.06) was observed. After adjusting for potential confounding factors (ECOG PS, age, gender, number of treatment lines, NLR, AFP, and LDH), OS was significantly prolonged in patients with RCCEP (adjusted HR 0.62, *p* = 0.04), whereas there was no statistically significant impact on PFS (adjusted HR 0.32, *p* = 0.72). Additionally, no statistically significant differences were found in the separate analysis of endocrine irAEs, hepatobiliary irAEs, and pulmonary irAEs. Due to the small sample size, enterocolitis and bladder irAEs were not analyzed individually.

### 3.4 Apatinib‐Related Specific AEs Profile

Among the 189 patients, 188 (99.5%) experienced apatinib‐related AEs. 139 (73.5%), 122 (64.6%) and 108 (57.1%) patients experienced any grade of apatinib‐related HTN, proteinuria, and HFS, respectively. The median onset times for these AEs were 1.3, 6.0, and 2.1 weeks, respectively. The incidences of grade 3–4 AEs mentioned above were 34.4%, 6.9%, and 9.0%, respectively. No grade 5 apatinib‐related AEs occurred.

### 3.5 Correlation Between Apatinib‐Related Specific AEs and Efficacy

**FIGURE 5:**
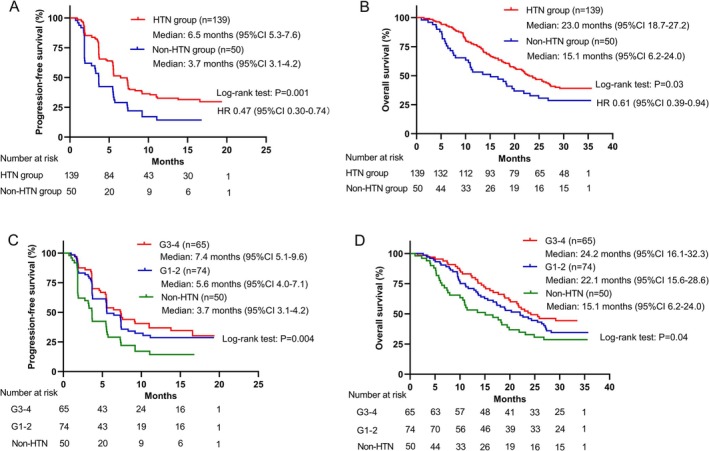
Comparisons in survival between the HTN and Non‐HTN groups. Kaplan–Meier curves of (A) progression‐free survival and (B) overall survival. Kaplan–Meier curves of (C) progression‐free survival and (D) overall survival for patients with grade 3–4 HTN.

The correlation analysis between apatinib‐related specific AEs and tumor response is shown in Tables S3 and S4. The occurrence of apatinib‐related HTN was significantly associated with increased ORR (38.1% vs. 18.0%, *p* = 0.009) and DCR (84.2% vs. 60.0%, *p* < 0.001), as well as prolonged PFS (6.5 vs. 3.7 months, *p* = 0.001) and OS (23.0 vs. 15.1 months, *p* = 0.03) (Figure 5A,B). Consistent results were observed in both first‐line and second‐line patients (Figure S5A–D, Tables S3 and S4). There were no statistically significant differences in TTR (1.9 vs. 1.8 months, *p* = 0.24) and DOR (9.3 vs. 7.9 months, *p* = 0.59) between patients with and without apatinib‐related HTN. Patients who experienced grade 3–4 HTN had a numerically longer median PFS and OS compared to those with grade 1–2 HTN or without HTN (Figure 5C,D). Since the median onset time of HTN was 1.3 weeks, much earlier than the time of progression or death, and 86% of the HTN events occurred prior to the first tumor assessment, no time‐dependent analysis was conducted to explore the correlation between HTN and efficacy.

## 4 Discussion

We described the profiles of irAEs and apatinib‐related specific AEs and evaluated their correlation with treatment efficacy in a multi‐center phase II trial involving patients with uHCC treated with a combination of camrelizumab and apatinib. Our results revealed that patients who experienced irAEs exhibited a better tumor response, although there was no significant difference in survival between the irAE group and Non‐irAE group. Additionally, patients who experienced apatinib‐related HTN showed a better clinical outcome.

ICIs enhance the activity of the immune system to exert anti‐tumor effects but also cause inflammatory side effects, commonly known as irAEs, which often involve the skin, lung, gastrointestinal tract, liver, endocrine system, and other organs. The overall incidence of any grade irAEs ranges from 54% to 76%, and the occurrence time is related to the involved organ system, typically occurring 2 to 16 weeks after the initiation of treatment [28]. The precise pathophysiology of irAEs remains unclear. It is currently believed to be related to the role of immune checkpoints in maintaining immunologic homeostasis and is caused by excessive immune activity against normal organs [29]. In our study, we found that the incidence and occurrence time of irAEs significantly differed from those observed with monotherapy when combined with anti‐angiogenic targeted therapy. This difference mainly manifested as a decrease in incidence, both for any grade (irAE: 46.6% vs. 80.0%; RCCEP: 29.6% vs. 67%) and for grade ≥ 3 (irAE: 9.5% vs. 22%), as well as a delay in occurrence time (RCCEP: 22.9 vs. 4.1 weeks) [30]. Mechanistically, anti‐angiogenic drugs can improve the immunosuppressive microenvironment characterized by tumor‐associated hypoxia, acidity, and high interstitial pressure resulting from local vascular abnormalities. This improvement contributes to the transport of immunotherapy agents and immune effector cells to the tumor site, enhancing anti‐tumor effects and reducing the systemic reactions [31]. Regarding RCCEP, it has been reported that camrelizumab acts as a potent agonist of human vascular endothelial growth factor receptor 2 (VEGFR‐2), leading to the occurrence of RCCEP [32]. Therefore, we infer that apatinib, a highly selective VEGFR‐2 inhibitor, can significantly reduce the incidence of RCCEP.

In our study, although there were differences in efficacy between first‐line and second‐line patients, their correlation with TRAEs was similar. Furthermore, the distribution of first‐line and second‐line patients was balanced in the irAE group and Non‐irAE group (Table S1), and there were no differences in the incidence (49.3% vs. 45.0%, *p* = 0.57) and median onset time (16.1 vs. 18.0 weeks, *p* = 0.52) of irAEs between first‐line and second‐line patients. Therefore, it was reasonable to perform analyses in the overall population. Our data showed that the occurrence of irAEs was associated with a better tumor response in patients with uHCC treated with the combination of camrelizumab and apatinib (ORR: 45.5% vs. 21.8%, *p* = 0.001; DCR: 87.5% vs. 69.3%, *p* = 0.003), which was consistent with several previous studies conducted in patients with advanced solid tumors [21, 22, 23, 33]. This could be attributed to higher T cell activity in patients experiencing irAEs [34], resulting in a more active anti‐tumor effect and an increased possibility of tumor response. There was no difference in TTR between the two groups (1.87 vs. 1.84 months, *p* = 0.34), indicating that the high response rate in the irAE group was not influenced by the duration of drug exposure. Thus, the bias of treatment duration can be excluded to support our findings. However, when considering the timing of irAE occurrence and tumor response, irAE cannot be regarded as a predictor of tumor response as it occurred later than tumor response in 75.0% of patients.

In this study, patients with irAEs showed a significant increase in median PFS (7.4 vs. 3.8 months, *p* < 0.001) and median OS (26.8 vs. 17.7 months, *p* = 0.002) compared to those without irAE. Nevertheless, after accounting for immortal time bias, no statistical differences in PFS and OS were observed between the two groups in the 6‐ and 20‐week landmark analyses. Similarly, there were no significant PFS and OS prolongations observed in patients with irAEs in the time‐dependent Cox regression analysis, which has been shown to be superior to landmark analysis in minimizing immortal time bias [35, 36]. Taking into account the unique nature of camrelizumab‐related RCCEP, we performed the same analysis on irAEs excluding RCCEP and found no correlation between the occurrence of irAEs excluding RCCEP and survival outcomes. However, our results contradicted the findings of two studies conducted in advanced HCC patients receiving ICIs monotherapy [22, 37]. In Lu et al.'s study, the occurrence of irAEs (especially rash) was associated with a significant improvement in PFS [22]; however, this study did not account for immortal time bias, which could lead to the opposite conclusion [38]. Ng et al. used landmark analysis to minimize time bias and found that the occurrence of irAEs was associated with significantly longer PFS and OS [37]. Compared to our study, the onset time of irAEs was earlier (dermatological events occurred after 4 weeks; hepatobiliary events occurred after 3.9 weeks; gastrointestinal events occurred after 4.4 weeks) in Ng et al.'s study [37], which may explain the inconsistent results.

In the analysis aimed at eliminating time bias, we observed that a significant number of patients who experienced irAEs after 6 weeks of treatment were included in the Non‐irAE group during the 6‐week landmark analysis. This was due to the delayed occurrence of irAEs in patients receiving combination therapy. Conversely, a considerable number of patients who experienced tumor progression before 20 weeks were excluded from the 20‐week landmark analysis, potentially compromising the statistical power of the analysis. In the time‐dependent Cox analysis, patients were included in the Non‐irAE group prior to the occurrence of irAEs. Due to the delayed onset of irAEs, the PFS and OS in the Non‐irAE group were artificially extended, which may explain the lack of statistical significance. This suggests that as irAEs occur later, it becomes more challenging to distinguish whether they are due to prolonged medication duration or not. Therefore, we conclude that irAEs cannot predict survival outcomes in our study, considering the decreased incidence and delayed occurrence time.

HTN, proteinuria, and HFS are common side effects associated with anti‐angiogenic drugs targeting the VEGF pathway. The exact mechanisms underlying these effects have not been fully elucidated, but several studies have indicated their association with VEGF pathway inhibition in non‐tumor cells [26]. Compared to monotherapy, the incidence of apatinib‐related AEs was increased (HTN: 73.5% vs. 48%; proteinuria: 64.6% vs. 44%; HFS: 57.1% vs. 56%) [39], and their time of occurrence did not change obviously after combining with PD‐1 blockade. Mechanistically, ICIs improve the tumor microenvironment and promote the normalization of tumor vasculature through a variety of mechanisms [40]. When combined with anti‐angiogenic drugs, ICIs enhance the mechanism of action of the latter, amplifying their effect and increasing the incidence of anti‐angiogenesis‐related toxicity. In accordance with the results of anti‐angiogenesis monotherapy, we found that patients experiencing apatinib‐related HTN in combination therapy also exhibited improved tumor response (ORR: 38.1% vs. 18.0%, *p* = 0.009; DCR: 84.2% vs. 60.0%, *p* < 0.001) and survival outcomes (PFS: 6.5 vs. 3.7 months, *p* = 0.001; OS: 23.0 vs. 15.1 months, *p* = 0.03). Therefore, we believe that the incidence of apatinib‐related HTN can still serve as a predictor of efficacy in our study, considering the increased incidence and similar occurrence time compared to apatinib monotherapy.

It is important to acknowledge certain limitations of this study. First, some TRAEs in the combination therapy could not be specifically classified as apatinib‐related or camrelizumab‐related, which may have influenced the results. Second, irAEs were considered within a 90‐day timeframe after the last dose, potentially overlooking irAEs occurring beyond this time window. Third, the sample size remained small, necessitating larger real‐world studies or prospective randomized controlled trials to further explore the relationship between irAEs and efficacy in combination therapy.

In conclusion, our findings suggest that irAEs cannot predict the efficacy in patients with uHCC treated with camrelizumab plus apatinib, owing to the decreased incidence and delayed occurrence of irAEs. On the other hand, apatinib‐related HTN shows promise as a potential predictor of efficacy, given its increased incidence and similar occurrence time compared to monotherapy.

## Author Contributions

**Ting Zhang:** conceptualization (equal), data curation (equal), formal analysis (equal), methodology (equal), writing – original draft (equal). **Sicheng Du:** formal analysis (equal), methodology (equal), writing – original draft (equal). **Ying Zhang:** formal analysis (equal), writing – original draft (equal). **Rongrui Liu:** conceptualization (equal), methodology (equal), supervision (equal). **Juan Li:** conceptualization (equal), methodology (equal), supervision (equal). **Chuanhua Zhao:** conceptualization (equal), methodology (equal), project administration (equal), supervision (equal), writing – review and editing (equal). **Jianming Xu:** conceptualization (equal), project administration (equal), resources (equal), supervision (equal), writing – review and editing (equal).

## Ethics Statement

This study was approved by the following ethics committees: Institutional Review Board for Drug Clinical Trials of the Fifth Medical Center of Chinese PLA General Hospital; Institutional Review Board of Tianjin Medical University Cancer Institute and Hospital; Institutional Review Board of Harbin Medical University Cancer Hospital; Institutional Review Board of the First Affiliated Hospital of Anhui Medical University; Institutional Review Board of Jiangsu Cancer Hospital Drug Clinical Trial Institution; Institutional Review Board of Nanjing Drum Tower Hospital, the Affiliated Hospital of Nanjing University Medical School; Institutional Review Board of Zhongshan Hospital, Fudan University; Institutional Review Board of the First Affiliated Hospital, Zhejiang University School of Medicine; Institutional Review Board of Zhejiang Cancer Hospital; Institutional Review Board of the First Affiliated Hospital of Nanchang University; Institutional Review Board of Hubei Cancer Hospital Ethics Office; Institutional Review Board of Xiangya Hospital, Central South University; Institutional Review Board of Hunan Provincial People's Hospital; Institutional Review Board of Hunan Cancer Hospital; Institutional Review Board of West China Hospital, Sichuan University; Institutional Review Board of the First Affiliated Hospital of Third Military Medical University; Institutional Review Board of the First Affiliated Hospital of Xi'an Jiaotong University; Institutional Review Board of the First Affiliated Hospital of Fourth Military Medical University; Institutional Review Board of Henan Cancer Hospital; Institutional Review Board of the First Affiliated Hospital of Zhengzhou University; Clinical Trial Institution/Institutional Review Board of the 900 Hospital of Joint Logistic Support Force of People's Liberation Army; Institutional Review Board of Mengchao Hepatobiliary Hospital of Fujian Medical University Ethics Office; Institutional Review Board of Sun Yat‐sen University Cancer Center Clinical Trial Institution; Institutional Review Board of Guangdong Provincial People's Hospital; and Institutional Review Board of Guangxi Medical University Affiliated Tumor Hospital. This study was carried out in accordance with the Declaration of Helsinki and the International Conference on Good Clinical Practice Standards.

## Consent

All patients provided written informed consent.

## Conflicts of Interest

The authors declare no conflicts of interest.

## Supporting information


Data S1.

## Data Availability

Data are available upon reasonable request. The data generated in this study are available on request from the corresponding author, with the permission of Jiangsu Hengrui Pharmaceuticals Co. Ltd.
