# Supplementary material for: Correlation Between Treatment‐Related Adverse Events and Efficacy of Camrelizumab in Combination With Apatinib in Patients With Unresectable Hepatocellular Carcinoma
Source: Cancer Med. 2025 Mar 23;14(6):e70713. doi: 10.1002/cam4.70713 (PMC11930853; doi:10.1002/cam4.70713)
Supplement: Supplementary file 1 — Data S1. [file CAM4-14-e70713-s001.docx]

Supplementary Table 1. Clinical characteristics of patients in the irAE group and non-irAE group

|  | Total  (n = 189) | irAE group  (*n*=91) | Non-irAE group  (*n*=98) | *P* value |
| --- | --- | --- | --- | --- |
| Age |  |  |  | 0.53 |
| <60 years | 145(76.7) | 68(74.7) | 77(78.6) |  |
| ≥60 years | 44(23.3) | 23(25.3) | 21(21.4) |  |
| Gender |  |  |  | 0.77 |
| Male | 169(89.4) | 82(90.1) | 87(88.8) |  |
| Female | 20(10.6) | 9(9.9) | 11(11.2) |  |
| Number of treatment lines |  |  |  | 0.40 |
| First-line | 69(36.5) | 36(39.6) | 33(33.7) |  |
| Second-line | 120(63.5) | 55(60.4) | 65(66.3) |  |
| ECOG PS |  |  |  | 0.97 |
| 0 | 114(60.3) | 55(60.4) | 59(60.2) |  |
| 1 | 75(39.7) | 36(39.6) | 39(39.8) |  |
| Child-Pugh score |  |  |  | 0.15 |
| A5 | 161(85.2) | 81(89.0) | 80(81.6) |  |
| A6 | 28(14.8) | 10(11.0) | 18(18.4) |  |
| BCLC stage |  |  |  | 0.32 |
| B | 34(18.0) | 19(20.9) | 15(15.3) |  |
| C | 155(82.0) | 72(79.1) | 83(84.7) |  |
| Extrahepatic metastases | 149(78.8) | 67(73.6) | 82(83.7) | 0.09 |
| Macrovascular invasion | 49(25.9) | 20(22.0) | 29(29.6) | 0.23 |
| HBV infection | 167(88.4) | 77(84.6) | 90(91.8) | 0.12 |
| AFP |  |  |  | 0.53 |
| <400 ng/mL | 94(49.7) | 48(52.7) | 46(46.9) |  |
| ≥400 ng/mL | 92(48.7) | 41(45.1) | 51(52.0) |  |
| Unknown | 3(1.6) | 2(2.2) | 1(1.0) |  |
| NLR |  |  |  | 0.80 |
| <3 | 125(66.1) | 61(67.0) | 64(65.3) |  |
| ≥3 | 64(33.9) | 30(33.0) | 34(34.7) |  |
| Platelet count |  |  |  | 0.63 |
| <130×10^9^ /L | 55(29.1) | 28(30.8) | 27(27.6) |  |
| ≥130×10^9^ /L | 134(70.9) | 63(69.2) | 71(72.4) |  |
| LDH |  |  |  | 0.03 |
| ≤1 ULN | 152(80.4) | 79(86.8) | 73(74.5) |  |
| >1 ULN | 37(19.6) | 12(13.2) | 25(25.5) |  |
| PD-L1 expression |  |  |  | 0.94 |
| TPS <1% | 32(16.9) | 15(16.5) | 17(17.3) |  |
| TPS ≥1% | 22(11.6) | 10(11.0) | 12(12.2) |  |
| Unknown | 135(71.4) | 66(72.5) | 69(70.4) |  |
| TMB |  |  |  | 0.14 |
| TMB-H | 21(11.1) | 8 (8.8) | 13(13.3) |  |
| TMB-L | 90(47.6) | 50(54.9) | 40(40.8) |  |
| Unknown | 78(41.3) | 33(36.3) | 45(45.9) |  |
| MSI |  |  |  | 0.65 |
| MSS | 43(22.8) | 22(24.2) | 21(21.4) |  |
| Unknown | 146(77.2) | 69(75.8) | 77(78.6) |  |
| Prior local therapy |  |  |  |  |
| Surgery | 146(77.2) | 73(80.2) | 73(74.5) | 0.35 |
| Radiotherapy | 44(23.3) | 22(24.2) | 22(22.4) | 0.77 |
| Interventional therapy | 136(72.0) | 64(70.3) | 72(73.5) | 0.63 |
| Local ablation | 63(33.3) | 26(28.6) | 37(37.8) | 0.18 |

irAE, Immune-related adverse event; ECOG PS, Eastern Cooperative Oncology Group Performance Status; BCLC, Barcelona Clinic Liver Cancer; HBV, Hepatitis B virus; AFP, A-fetoprotein; NLR, Neutrophil to lymphocyte ratio; LDH, Lactate dehydrogenase; TPS, Tumour proportion score; TMB, Tumor mutational burden; TMB-H, Tumor mutational burden-high; TMB-L, Tumor mutational burden-low; MSI, Microsatellite instability; MSS, Microsatellite stability

Supplementary Table 2. Clinical characteristics of patients in the irAE (excluding RCCEP) group, Non-irAE (excluding RCCEP) group, RCCEP group and Non-RCCEP group.

|  | Total  (n = 189) | irAE (excluding RCCEP) group  (*n*=58) | Non-irAE (excluding RCCEP) group  (*n*=131) | *P* value | RCCEP group  (*n*=56) | Non-RCCEP group  (*n*=133) | *P* value |
| --- | --- | --- | --- | --- | --- | --- | --- |
| Age |  |  |  | 0.85 |  |  | 0.06 |
| <60 years | 145(76.7) | 45(77.6) | 100(76.3) |  | 38(67.9) | 107(80.5) |  |
| ≥60 years | 44(23.3) | 13(22.4) | 31(23.7) |  | 18(32.1) | 26(19.5) |  |
| Gender |  |  |  | 0.94 |  |  | 0.58 |
| Male | 169(89.4) | 52(89.7) | 117(89.3) |  | 49(87.5) | 120(90.2) |  |
| Female | 20(10.6) | 6(10.3) | 14(10.7) |  | 7(12.5) | 13(9.8) |  |
| Number of treatment lines |  |  |  | 0.36 |  |  | 0.42 |
| First-line | 69(36.5) | 24(41.4) | 45(34.4) |  | 18(32.1) | 51(38.3) |  |
| Second-line | 120(63.5) | 34(58.6) | 86(65.6) |  | 38(67.9) | 82(61.7) |  |
| ECOG PS |  |  |  | 0.52 |  |  | 0.47 |
| 0 | 114(60.3) | 33(56.9) | 81(61.8) |  | 36(64.3) | 78(58.6) |  |
| 1 | 75(39.7) | 25(43.1) | 50(38.2) |  | 20(35.7) | 55(41.4) |  |
| Child-Pugh score |  |  |  | 0.25 |  |  | 0.30 |
| A5 | 161(85.2) | 52(89.7) | 109(83.2) |  | 50(89.3) | 111(83.5) |  |
| A6 | 28(14.8) | 6(10.3) | 22(16.8) |  | 6(10.7) | 22(16.5) |  |
| BCLC stage |  |  |  | 0.82 |  |  | 0.98 |
| B | 34(18.0) | 11(19.0) | 23(17.6) |  | 10(17.9) | 24(18.0) |  |
| C | 155(82.0) | 47(81.0) | 108(82.4) |  | 46(82.1) | 109(82.0) |  |
| Extrahepatic metastases | 149(78.8) | 41(70.7) | 108(82.4) | 0.07 | 43(76.8) | 106(79.7) | 0.65 |
| Macrovascular invasion | 49(25.9) | 16(27.6) | 33(25.2) | 0.73 | 9(16.1) | 40(30.1) | 0.05 |
| HBV infection | 167(88.4) | 49(84.5) | 118(90.1) | 0.27 | 46(82.1) | 121(91.0) | 0.08 |
| AFP |  |  |  | 0.89 |  |  | 0.20 |
| <400 ng/mL | 94(49.7) | 30(51.7) | 64(48.9) |  | 30(53.6) | 64(48.1) |  |
| ≥400 ng/mL | 92(48.7) | 27(46.6) | 65(49.6) |  | 24(42.8) | 68(51.1) |  |
| Unknown | 3(1.6) | 1(1.7) | 2(1.5) |  | 2(3.6) | 1(0.8) |  |
| NLR |  |  |  | 0.59 |  |  | 0.73 |
| <3 | 125(66.1) | 40(69.0) | 85(64.9) |  | 36(64.3) | 89(66.9) |  |
| ≥3 | 64(33.9) | 18(31.0) | 46(35.1) |  | 20(35.7) | 44(33.1) |  |
| Platelet count |  |  |  | 0.32 |  |  | 0.81 |
| <130×10^9^ /L | 55(29.1) | 14(24.1) | 41(31.3) |  | 17(30.4) | 38(28.6) |  |
| ≥130×10^9^ /L | 134(70.9) | 44(75.9) | 90(68.7) |  | 39(69.6) | 95(71.4) |  |
| LDH |  |  |  | 0.03 |  |  | 0.23 |
| ≤1 ULN | 152(80.4) | 52(89.7) | 100(76.3) |  | 48(85.7) | 104(78.2) |  |
| >1 ULN | 37(19.6) | 6(10.3) | 31(23.7) |  | 8(14.3) | 29(21.8) |  |
| PD-L1 expression |  |  |  | 0.52 |  |  | 0.07 |
| TPS <1% | 32(16.9) | 10(17.2) | 22(16.8) |  | 9(16.1) | 23(17.3) |  |
| TPS ≥1% | 22(11.6) | 9(15.5) | 13(9.9) |  | 2(3.6) | 20(15.0) |  |
| Unknown | 135(71.4) | 39(67.2) | 96(73.3) |  | 45(80.3) | 90(67.7) |  |
| TMB |  |  |  | 0.07 |  |  | 0.60 |
| TMB-H | 21(11.1) | 5 (8.6) | 16(12.2) |  | 7(12.5) | 14(10.5) |  |
| TMB-L | 90 (47.6) | 35(60.3) | 55(42.0) |  | 29(51.8) | 61(45.9) |  |
| Unknown | 78(41.3) | 18(31.0) | 60 (45.8) |  | 20(35.7) | 58(43.6) |  |
| MSI |  |  |  | 0.15 |  |  | 0.51 |
| MSS | 43(22.8) | 17(29.3) | 26(19.8) |  | 11(19.6) | 32(24.1) |  |
| Unknown | 146(77.2) | 41(70.7) | 105(80.2) |  | 45(80.4) | 101(75.9) |  |
| Prior local therapy |  |  |  |  |  |  |  |
| Surgery | 146(77.2) | 48(82.8) | 98(74.8) | 0.23 | 45(80.4) | 101(75.9) | 0.51 |
| Radiotherapy | 44(23.3) | 14(24.1) | 30(22.9) | 0.85 | 12(21.4) | 32(24.1) | 0.70 |
| Interventional therapy | 136(72.0) | 42(72.4) | 94(71.8) | 0.93 | 39(69.6) | 97(72.9) | 0.65 |
| Local ablation | 63(33.3) | 14(24.1) | 49(37.4) | 0.07 | 19(33.9) | 44(33.1) | 0.91 |

RCCEP, Reactive cutaneous capillary endothelial proliferation

Supplementary Table 3. Correlation between tumor response and apatinib-related sepcific AEs in 189 patients treated with camrelizumab and apatinib per mRECIST

| mRECIST | Hypertension | | P value | Proteinuria | | P value | Hand-foot syndrome | | P value |
| --- | --- | --- | --- | --- | --- | --- | --- | --- | --- |
|  | Yes | No |  | Yes | No |  | Yes | No |  |
| ORR, % | 38.1 | 18.0 | **0.009** | 37.7 | 23.9 | 0.05 | 31.5 | 34.6 | 0.66 |
| First-line | 49.0 | 38.9 | 0.46 | 55.6 | 29.2 | **0.04** | 45.2 | 47.4 | 0.86 |
| Second-line | 31.8 | 6.3 | **0.009** | 27.3 | 20.9 | 0.44 | 26.0 | 23.3 | 0.74 |
| DCR, % | 84.2 | 60.0 | **<0.001** | 82.0 | 70.1 | 0.06 | 83.3 | 70.4 | **0.03** |
| First-line | 88.2 | 61.1 | **0.01** | 91.1 | 62.5 | **0.01** | 93.5 | 71.1 | **0.04** |
| Second-line | 81.8 | 59.4 | **0.01** | 76.6 | 74.4 | 0.79 | 79.2 | 69.8 | 0.25 |

AEs, Adverse events; mRECIST, Modified Response Evaluation Criteria In Solid Tumors; ORR, Objective response rate; DCR, Disease control rate

Supplementary Table 4. Correlation between tumor response and apatinib-related sepcific AEs in 189 patients treated with camrelizumab and apatinib per RECIST v1.1

| RECIST v1.1 | Hypertension | | P value | Proteinuria | | P value | Hand-foot syndrome | | P value |
| --- | --- | --- | --- | --- | --- | --- | --- | --- | --- |
|  | Yes | No |  | Yes | No |  | Yes | No |  |
| ORR, % | 30.9 | 16.0 | **0.04** | 30.3 | 20.9 | 0.16 | 25.9 | 28.4 | 0.71 |
| First-line | 37.3 | 27.8 | 0.47 | 37.8 | 29.2 | 0.47 | 35.5 | 34.2 | 0.91 |
| Second-line | 27.3 | 9.4 | **0.07** | 26.0 | 16.3 | 0.22 | 22.1 | 23.3 | 0.88 |
| DCR, % | 84.2 | 58.0 | **<0.001** | 81.1 | 70.1 | 0.08 | 84.3 | 67.9 | **0.008** |
| First-line | 88.2 | 55.6 | **0.003** | 91.1 | 58.3 | **0.004** | 93.5 | 68.4 | **0.02** |
| Second-line | 81.8 | 59.4 | **0.01** | 75.3 | 76.7 | 0.86 | 80.5 | 67.4 | 0.11 |

RECIST, Response Evaluation Criteria In Solid Tumors


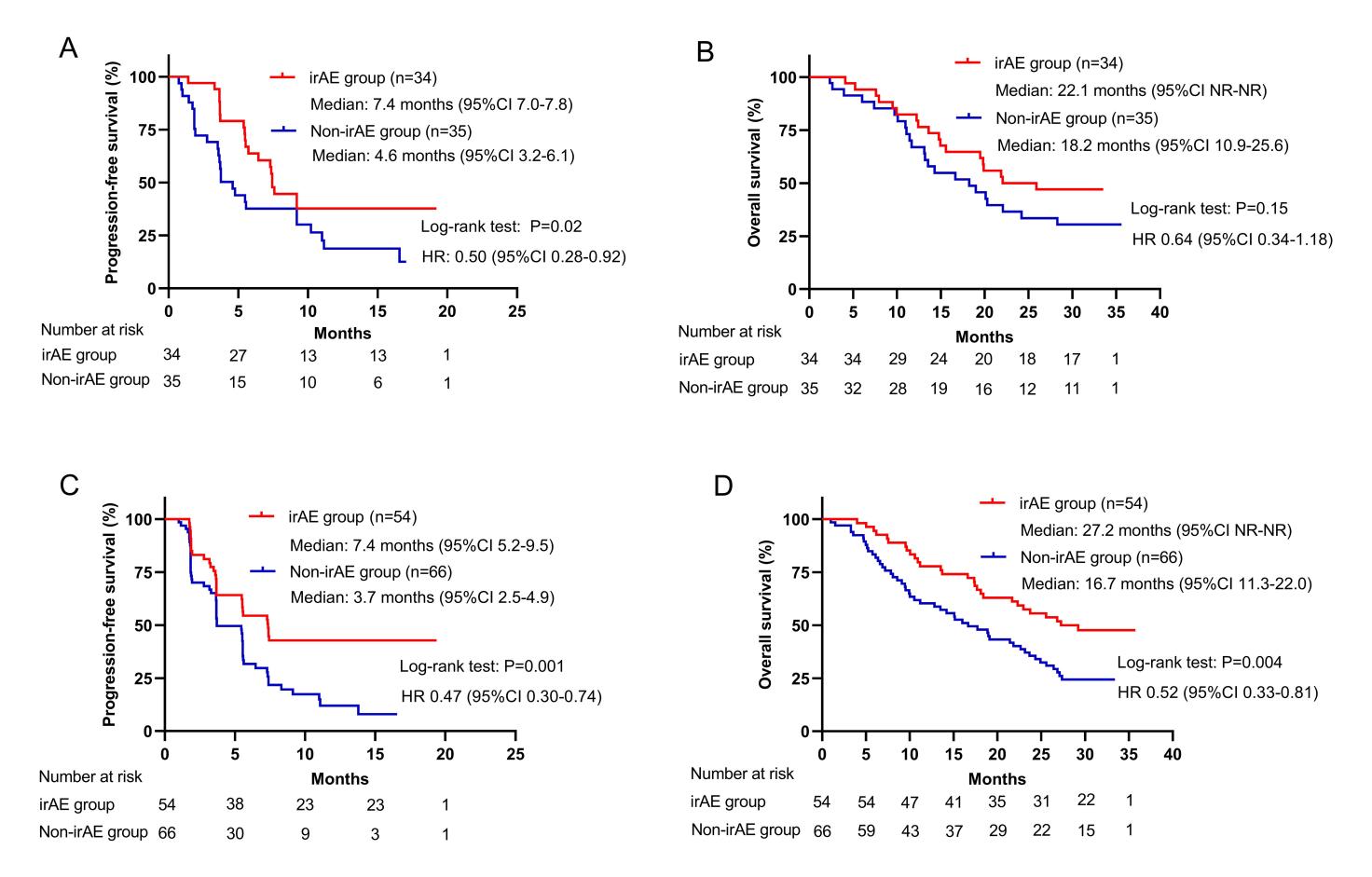


Supplementary figure 1. Comparisons in survival between irAE group and Non-irAE group in first-line and second-line patients. Kaplan-Meier curves of (A) progression-free survival and (B) overall survival in first-line patients. Kaplan-Meiers curve of (C) progression-free survival and (D) overall survival in second-line patients.


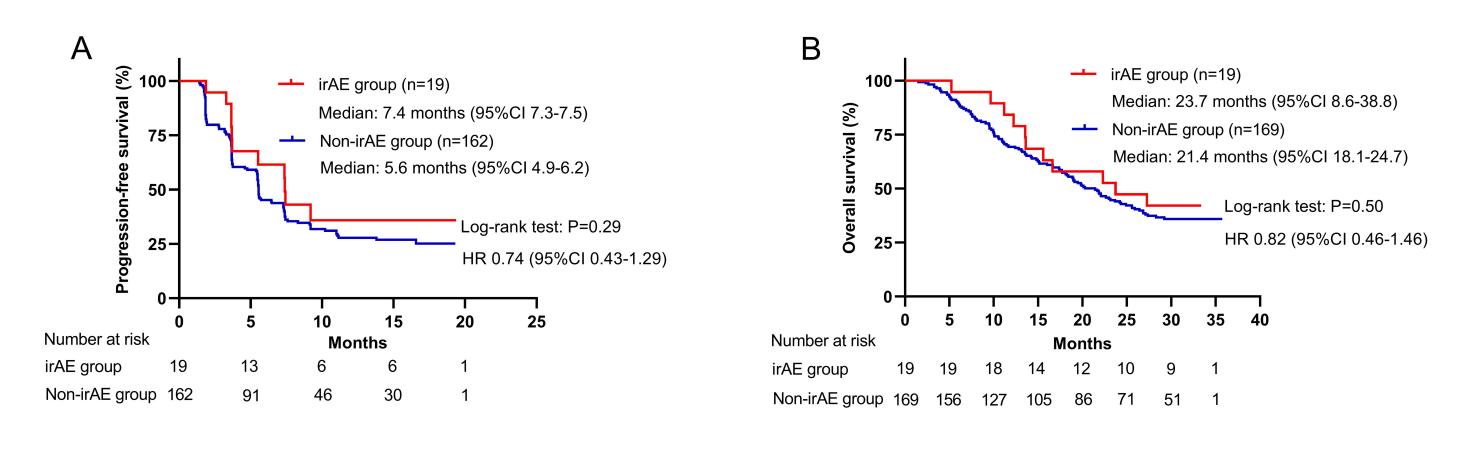


Supplementary figure 2. Kaplan-Meier curves with 6-week landmark analysis for (A) progression-free survival and (B) overall survival in patients with and without irAE.


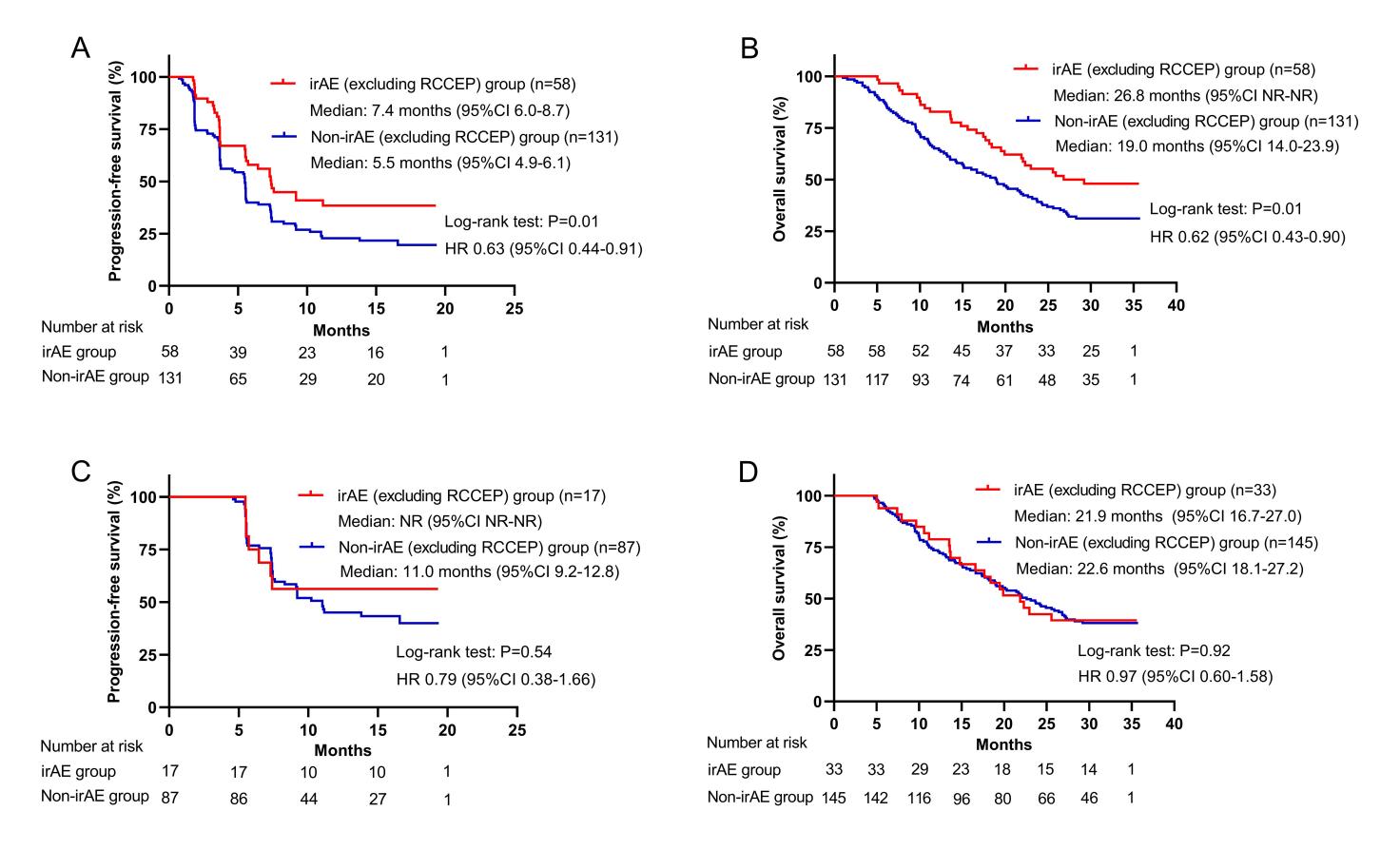


Supplementary figure 3. Comparisons in survival between irAE (excluding RCCEP) group and Non-irAE (excluding RCCEP) group. Kaplan-Meier curves of (A) progression-free survival and (B) overall survival. Kaplan-Meier curves with 20-week landmark analysis for (C) progression-free survival and (D) overall survival.


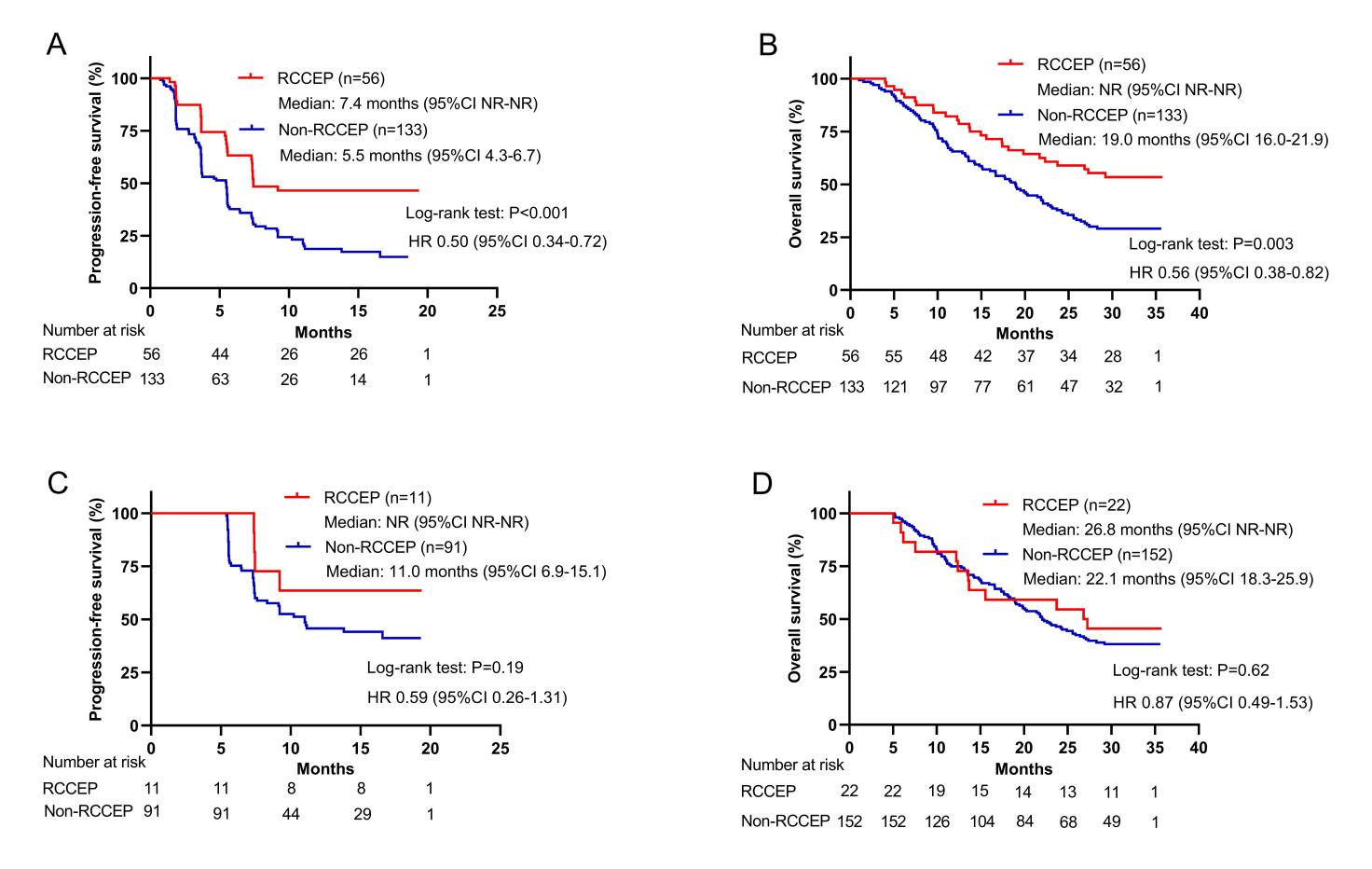


Supplementary figure 4. Comparisons in survival between RCCEP group and Non-RCCEP group. Kaplan-Meier curves of (A) progression-free survival and (B) overall survival. Kaplan-Meier curves with 20-week landmark analysis for (C) progression-free survival and (D) overall survival.


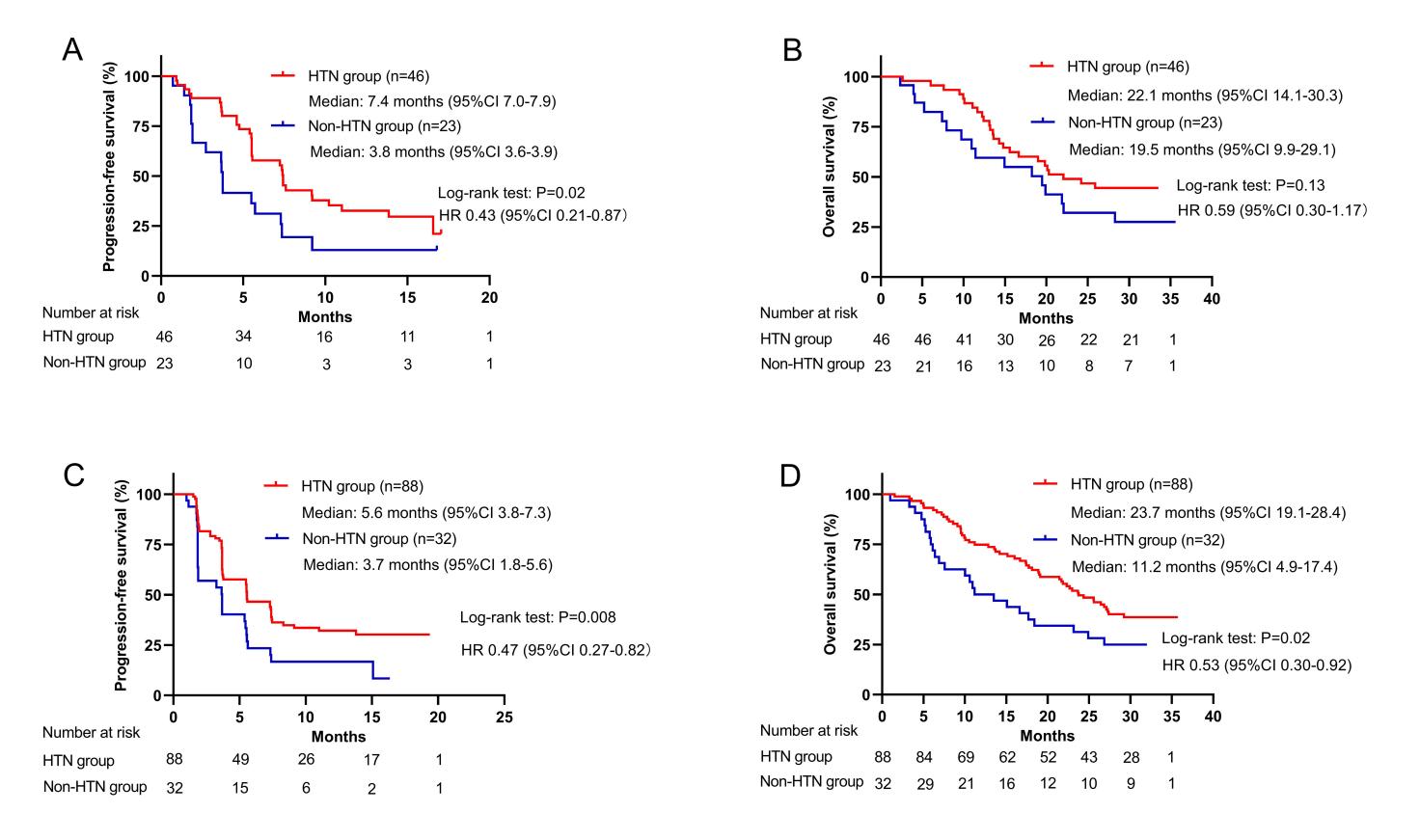


Supplementary figure 5. Comparisons in survival between HTN group and Non-HTN group in first-line and second-line patients. Kaplan-Meier curves of (A) progression-free survival and (B) overall survival in first-line patients. Kaplan-Meier curves of (C) progression-free survival and (D) overall survival in second-line patients.
